# Changes in Infant and Neonatal Mortality and Associated Factors in Eight Cohorts from Three Brazilian Cities

**DOI:** 10.1038/s41598-020-59910-7

**Published:** 2020-02-24

**Authors:** Carolina A. Carvalho, Antônio A. M. da Silva, César Victora, Marcelo Goldani, Heloísa Bettiol, Erika Barbara Thomaz, Fernando Barros, Bernardo L. Horta, Ana Menezes, Viviane Cardoso, Ricardo Carvalho Cavalli, Iná Santos, Rosângela F. L. Batista, Vanda Maria Simões, Marco Barbieri, Aluisio Barros

**Affiliations:** 1 Federal Institute of Maranhão; Federal University of Maranhão, Post Graduate Program in Collective Health, Rua Barão de Itapary, nº 155, Centro, Zipcode: 65.020-070, São Luís, MA Brazil; 2 Federal University of Maranhão, Post Graduate Program in Collective Health, Rua Barão de Itapary, nº 155, Centro, Zipcode: 65.020-070, São Luís, MA Brazil; 3 Federal University of Pelotas, Post Graduate Program in Epidemiology, Pelotas, Brazil; 4 Federal University of Rio Grande do Sul, Department of Pediatrics, Porto Alegre, Brazil; 5 University of São Paulo, Department of Puericulture and Pediatrics, Ribeirão Preto, Brazil; 6 Catholic University of Pelotas, Post-Graduate Program in Health and Behavior and Federal University of Pelotas, Post Graduate Program in Epidemiology, Pelotas, Brazil; 7 University of São Paulo, Department of Gynecology and Obstetrics, Ribeirão Preto, Brazil

**Keywords:** Neonatology, Risk factors

## Abstract

Stillbirth (SBR), perinatal (PMR), neonatal (NMR) and infant mortality rates (IMR) are declining in Brazil and the factors associated with these falls are still being investigated. The objective of the present study was to assess changes in SBR, PMR, NMR and IMR over time and to determine the factors associated with changes in NMR and IMR in eight Brazilian cohorts. All cohorts are population-based (Ribeirão Preto in 1978/79, 1994 and 2010; Pelotas in 1982, 1993 and 2004; and São Luís in 1997/98 and 2010). Were included data on 41440 children. All indicators were decreased, except in the city of Pelotas, from 1993 to 2004, and except SBR in São Luís. Sociodemographic variables seem to be able to explain reductions of NMR and IMR in Ribeirão Preto, from 1978/79 to 1994, and in São Luís. In Ribeirão Preto, from 1994 to 2010 declines in NMR and IMR seem to be explained by reductions in intrauterine growth restriction (IUGR). Newborn’s gestational age had diminished in all cohorts, preventing even greater reductions of NMR and IMR. Improved sociodemographic variables and reduction of IUGR, seem to be able to explain part of the decrease observed. NMR and IMR could have been reduced even more, were it not for the worsening in gestational age distribution.

## Introduction

The infant mortality rate (IMR) is considered to be one of the most relevant indicators of the living conditions of a population^1,2^. Stillbirths, perinatal mortality and neonatal mortality reflect factors related to the quality of prenatal and childbirth care, while the postnatal component is mainly related to socio-environmental factors^3,4^ and to the management of infant diseases.

Although the reduction of IMR and of perinatal mortality rate (PMR) is a worldwide trend^5–9^, some developed countries are showing stabilization or a significant reduction of the rate of fall^10–12^. In Brazil, over the last few years there has been a reduction of these indicators thanks to improved maternal and child health care, favourable changes in sociodemographic conditions, and a reduction of social inequality^13–16^. However, during more recent periods PMR has tended to show deceleration of reduction and even stabilization^16^. Neonatal mortality rate (NMR), the main component of IMR in Brazil, has been following a declining trend^17^.

Over the last 40 years, Brazil has been going through an intense process of epidemiologic and demographic transition. Although the national trend is of reduction, this occurs unequally in the various regions of the country and among the income quintiles^13^.

The magnitude of reduction in stillbirth rate (SBR), PMR, NMR and IMR and the factors associated with changes in infant mortality indicators in Brazil have not been fully elucidated. On this basis, the objective of our study was to assess changes in SBR, PMR, NMR and IMR and to determine some of the factors associated with changes in NMR and IMR in eight birth cohorts followed up in three Brazilian cities from different regions of the country. The analyses performed cover a period of great changes in sociodemographic and economic development of the country.

## Methods

The data were population-based from eight birth cohorts followed up in the Brazilian cities of Ribeirão Preto (1978/79, 1994 and 2010), Pelotas (1982, 1993 and 2004) and São Luís (1997/98 and 2010).

Ribeirão Preto, located in the state of São Paulo, Brazilian Southeast region, is one of the most developed cities in the country, with a human development index (HDI) of 0.800. All cohorts involved the evaluation of the births that occurred at all maternities. The first cohort included 7248 births from June 1, 1978 to May 29, 1979. A total of 3015 births were included in second birth cohort from April 25 to August 25, 1994. The third Ribeirão Preto birth cohort included 7798 births from January 1 to December 31, 2010, as part of the BRISA multicenter study (*Brazilian Birth Cohort Studies, Ribeirão Preto and São Luís*). Losses due to refusal or impossibility of locating the mother were <5%.

In Pelotas, a city with an HDI of 0.739 located in the state of Rio Grande do Sul, three birth cohorts were started in 1982, 1993 and 2004 using a similar methodology for all of them, with evaluation of infants born at all maternities in the city from January 1 to December 31 of the respective years. The total number of births was 6011 in 1982, 5304 in 1993 and 4287 in 2004. The refusal rate was less than 1% for each cohort.

São Luís is the capital city of the state of Maranhão, located in the Brazilian Northeast, one of the poorest regions in the country, with an HDI of 0.768. Data were collected for one in each seven births that occurred in the city from March 1, 1997 to February 28, 1998, totalling 2541 children. The second São Luís cohort included one in each three liveborn babies (5236) from January 1 to December 31, 2010, as part of the multicenter BRISA study. Losses due to refusal or impossibility of locating the mother were low, <4%.

In all cohorts the fetal and postnatal deaths were identified by linking cohort data with mortality registry data. Details about the methods of the Ribeirão Preto^18^, Pelotas^16,19^ and São Luís^14,18^ cohorts have been published previously.

Thus, data on a total of 41,440 children were included in the study. Data regarding socioeconomic and demographic variables, prenatal care and perinatal health were obtained using questionnaires applied to the mothers. Birth weight was measured shortly after delivery.

SBR was defined as the number of fetal deaths that occurred starting from the 22nd week of gestation divided by the total number of liveborns (LB) and stillbirths (SB) in the cities of Ribeirão Preto and São Luís. In Pelotas, SBR was defined as fetal deaths that occurred since the 28th week of gestation. PMR included stillbirths and early neonatal deaths (up to the first seven days after birth) divided by LB and SB. The NMR was characterized as the death of newborns from zero to 27 days of life completed and IMR as the deaths of infants younger than one year divided by the number of LB^9,20^.

Data were analysed using the Stata® software, version 14.0. The rates (per 1000 births) and confidence intervals for SBR, PMR, NMR and IMR were estimated. If the numbers of stillbirths or deaths were less than 100, 95% confidence intervals (95%CI) were estimated by Poisson distribution^10^.

An indicator variable called “time period” was created in order to represent the interval between one cohort and the other in the same city. Thus, two cohorts at a time were compared. For example, the time period indicator for Ribeirão Preto was coded as 0 for 1978/79 and as 1 for 1994. A second indicator variable was created for the same city comparing 1994 (coded as 0) to 2010 (coded as 1). This strategy was used because the reduction of infant and neonatal mortality differed according to the study period in each city, i.e., it was not linear.

**Figure 1:**
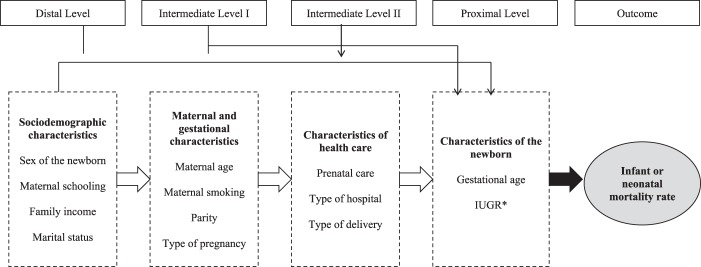
Hierarchical modelling for the analysis of factors associated with infant and neonatal mortality. *IUGR: Intrauterine growth restriction.

Logistic regression was used to determine the factors that explained the variation in NMR and IMR. We did not investigate the explanatory factors for SBR and PMR since not all São Luís and Ribeirão Preto cohorts had complete information about explanatory variables of fetal death cases. The crude effect of time period on NMR and IMR was first estimated, followed by the introduction of variables in the hierarchical regression analysis, with calculation of the odds ratio (OR) adjusted on the basis of the addition of each group of variables (Fig. 1). The variables within a level showing a p value of less than 0.10 were maintained when the variables of the subsequent level were included. The significance level of 0.10 was used because the objective was to identify potential predictor variables rather than to test a hypothesis^21^. It is important to mention that the scientific plausibility was considered during the variables inclusion.

In the hierarchical approach, the selection of variables to be included in the multivariate model is not made simply based on statistical associations but based on a conceptual model that establishes hierarchical relationships between the factors associated with the outcome. In this analysis, the effect of each variable is interpreted at the hierarchical level at which it appears for the first time. Analyzing the effect of a variable at levels other than the first at which it appears is inappropriate, as the effect is underestimated due to the presence of other mediating factors^22^.

In the distal level we included the *sociodemographic characteristics:* newborn sex, maternal schooling (0–4, 5–8, 9–11, and ≥12 years), family income (in tertiles), marital status (with or without a partner). In the intermediate level I we included the *maternal and gestational characteristics:* maternal age (<20, 20–34, and ≥35 years), maternal smoking during pregnancy (yes or no), parity including the current pregnancy (1, 2–4, ≥5 children) and type of pregnancy (single or multiple). In the intermediate level II we considered the *health care characteristics:* prenatal care (yes or no), hospital where delivery was performed (public or private) and type of delivery (vaginal or cesarean). And in the proximal level we considered the *characteristics of the newborn (NB)*. Gestational age was estimated according to the date of last menstruation (DLM) in São Luís and Pelotas (1982). In Pelotas, in 1993 and 2004 and in Ribeirão Preto in 2010^23^, gestational age was calculated by the best obstetric estimate, based primarily on ultrasound, and secondarily on the DLM. Intrauterine growth restriction (IUGR) was defined as the ratio between birth weight and expected birthweight given gestational age and sex below a certain threshold proposed by Kramer *et al*.^24^ and growth was categorized as not restricted (>0.85), mildly or moderately restricted (0.75–0.84) and severely restricted (<0.75).

Based on this conceptual hierarchical framework, the model 1 was adjusted for sociodemographic characteristics, the model 2 was adjusted for sociodemographic characteristics plus maternal and gestational characteristics, the model 3 was adjusted for sociodemographic plus maternal and gestational plus health care characteristics, the model 4 was adjusted for sociodemographic plus maternal and gestational plus health care and newborn characteristics; the model 5 was just adjusted for gestational age and the model 6 was adjusted for intrauterine growth restriction only.

For the older cohorts (Ribeirão Preto 1978/79 and 1994, Pelotas 1982 and 1993 and São Luís 1997/98) we obtained the approval of an Internal Committee of the respective Schools of Medicine (Ethics Committee of the University Hospital of the Federal University of Maranhão, Ethics Committee of the Hospital of the Clinics of the Federal University of São Paulo, Ethics Committee of the Federal University of Pelotas) and verbal informed maternal consent. All other cohorts were approved by the Research Ethics Committees of the teaching institutions to which they were linked and the mothers gave written informed consent before interview. Therefore, additional informed consent was obtained from all individual participants for whom identifying information is included in this article.

## Results

**Figure 2:**
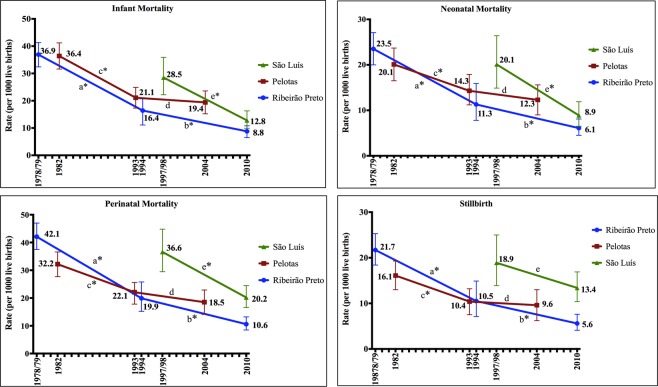
Changes in infant, neonatal, perinatal and stillbirth rates in Ribeirão Preto (1978/79, 1994 and 2010), Pelotas (1982, 1993 and 2004) and São Luís (1997/98 and 2010). *Indicates that the comparison of the mortality rate during the period showed a p < 0.05 value. (**a**) Comparison between the years 1978/79 and 1994; (**b**) Comparison between the years 1994 and 2010; (**c**) Comparison between the years 1982 and1993; (**d**) Comparison between the years 1993 and 2004; (**e)** Comparison between the years 1997/98 and 2010.

Most indicators were reduced in all cities, except Pelotas, from 1993 to 2004. However, SBR did not change in São Luís. The percentages of reduction were higher during the first periods studied (Fig. 2).

The Supplementary Tables 1 and 2 present changes in risk factors for IMR over time. Maternal smoking was reduced in all cities. Prenatal care utilization and use of private hospitals for delivery care increased in all comparisons except in Pelotas from 1982 to 1993. Maternal schooling and caesarean delivery increased in all three cities. Low maternal age tended to increase in the older periods and decrease in most recent periods, but in Pelotas it rose in all periods. Living without a companion increased in Ribeirão Preto and Pelotas but decreased in São Luís. Primiparity increased in Pelotas and in Ribeirão Preto but remained stable in São Luís. Mean gestational age was reduced from the 1^st^ to the 2^nd^ periods in Ribeirão Preto and Pelotas, and in São Luís. The prevalence of IUGR was increased from the 1^st^ to the 2^nd^ period in Ribeirão Preto but decreased thereafter, whereas in Pelotas it decreased from the 1^st^ to the 2^nd^ period but increased thereafter. In São Luís IUGR decreased.

In most of the unadjusted models – i.e. those for which the only explanatory variable was a dummy term for the cohort year - the OR were less than one, revealing a reduction of mortality along time. The variables that increased the OR in the adjusted models in relation to the crude OR were those that contributed to the explanation of the variations in NMR or IMR. In Ribeirão Preto, from the 1^st^ to the 2^nd^ period, there was an increase from 0.45 to 0.68 in the OR when sociodemographic characteristics were included in the model (Model 1, Table 1). Gestational age was the variable that led to the greater reduction in the OR (Model 5, Table 1). Similar results were observed in São Luís, with increased OR after inclusion of sociodemographic variables in the model and reduction of OR when the characteristics of newborns were included (Model 1 and 4, Table 1). From the 2^nd^ to the 3^rd^ period in Ribeirão Preto, there was an increase in the OR when IUGR variable was included (Model 6, Table 1). During this period, there was a reduction in the OR after the inclusion of sociodemographic characteristics in the model (Model 1, Table 1). In Pelotas, from the 1^st^ to the 2^nd^ period, the OR increased after the adjustment by IUGR variable, whereas the gestational age led to the reduction of OR (Model 5 and 6, Table 1).

**Table 1:** Hierarchical analysis of the association between year and infant mortality in Ribeirão Preto (1978/79, 1994 and 2010), Pelotas (1982, 1993 and 2004) ad São Luís (1997/98 and 2010).

	Ribeirão Preto		Pelotas		São Luís
	1978/79–1994	1994–2010	1982–1993	1993–2004	1997/98–2010
	OR (95% CI)	OR (95% CI)	OR (95% CI)	OR (95% CI)	OR (95% CI)
**Unadjusted**	**0.45 (0.33–0.62)**	**0.53 (0.36–0.76)**	**0.57 (0.45–0.72)**	0.92 (0.69–1.23)	**0.45 (0.32–0.63)**
**Model 1**	0.68 (0.45–1.04)	**0.32 (0.18–0.57)**	**0.52 (0.41–0.66)**	1.03 (0.76–1.40)	**0.56 (0.37–0.84)**
**Model 2**	0.68 (0.45–1.05)	**0.31 (0.18–0.56)**	**0.52 (0.41–0.66)**	0.99 (0.73–1.35)	**0.55 (0.37–0.82)**
**Model 3**	0.69 (0.43–1.10)	**0.27 (0.15–0.50)**	**0.52 (0.41–0.66)**	1.02 (0.74–1.40)	**0.57 (0.37–0.87)**
**Model 4**	**0.40 (0.24–0.67)**	**0.28 (0.15–0.56)**	**0.27 (0.20–0.38)**	0.85 (0.57–1.26)	**0.30 (0.18–0.50)**
**Model 5**	**0.29 (0.21–0.41)**	**0.40 (0.27–0.60)**	**0.30 (0.23–0.41)**	0.77 (0.54–1.09)	**0.32 (0.22–0.46)**
**Model 6**	**0.42 (0.31–0.58)**	**0.59 (0.40–0.86)**	**0.65 (0.51–0.84)**	0.93 (0.69–1.25)	**0.44 (0.31–0.62)**

OR: Odds ratio; CI: Confidence Interval. Values highlighted are those with significant statistical results.

**Model 1**: Adjusted for sociodemographic characteristics; **Model 2**: Adjusted for sociodemographic characteristics + maternal and gestational characteristics; **Model 3**: Adjusted for sociodemographic + maternal and gestational + health care characteristics; **Model 4**: Adjusted for sociodemographic + maternal and gestational + health care and newborn characteristics; **Model 5**: Adjusted for gestational age; **Model 6**: Adjusted for intrauterine growth restriction.

From 1978/79 to 1994 in Ribeirão Preto and from 1997/98 to 2010 in São Luís, there was an increase in the OR after the inclusion of the sociodemographic variables in the model (Model 1, Table 2). In Ribeirão Preto from 1994 to 2010 there was an increase in the OR after the inclusion of the IUGR variable and reduction of the OR with the addition of sociodemographic characteristics in the model (Model 6, Table 2). The adjustment for gestational age showed reduction in the OR in all cities (Model 5, Table 2).

**Table 2:** Hierarchical analysis of the association between year and neonatal mortality in Ribeirão Preto (1978/79, 1994 and 2010), Pelotas (1982, 1993 and 2004) ad São Luís (1997/98 and 2010).

	Ribeirão Preto		Pelotas		São Luís
	1978/79–1994	1994–2010	1982–1993	1993–2004	1997/98–2010
	OR (95% CI)	OR (95% CI)	OR (95% CI)	OR (95% CI)	OR (95% CI)
**Unadjusted**	**0.48 (0.33–0.70)**	**0.55 (0.35–0.86)**	**0.71 (0.53–0.94)**	0.86 (0.60–1.23)	**0.44 (0.30–0.66)**
**Model 1**	0.87 (0.53–1.45)	**0.31 (0.16–0.60)**	**0.66 (0.49–0.89)**	0.92 (0.63–1.34)	0.63 (0.39–1.02)
**Model 2**	0.88 (0.52–1.46)	**0.30 (0.15–0.57)**	**0.66 (0.48–0.89)**	0.88 (0.60–1.28)	0.62 (0.38–1.00)
**Model 3**	0.89 (0.50–1.57)	**0.26 (0.13–0.52)**	**0.66 (0.48–0.89)**	0.92 (0.62–1.35)	0.71 (0.43–1.18)
**Model 4**	**0.45 (0.23–0.88)**	**0.23 (0.11–0.51)**	**0.32 (0.20–0.51)**	**0.55 (0.31–0.98)**	**0.48 (0.27–0.86)**
**Model 5**	**0.29 (0.19–0.44)**	**0.37 (0.23–0.61)**	**0.31 (0.20–0.47)**	**0.55 (0.33–0.91)**	**0.35 (0.23–0.55)**
**Model 6**	**0.47 (0.32–0.69)**	**0.61 (0.38–0.96)**	0.84 (0.61–1.16)	0.91 (0.63–1.32)	**0.45 (0.29–0.68)**

OR: Odds ratio; CI: Confidence Interval. Values highlighted are those with significant statistical results.

**Model 1:** Adjusted for sociodemographic characteristics**; Model 2:** Adjusted for sociodemographic + maternal and gestational characteristics**; Model 3:** Adjusted for sociodemographic + maternal and gestational + health care characteristics; **Model 4:** Adjusted for sociodemographic + maternal and gestational + health care and newborn characteristics; **Model 5:** Adjusted for gestational age**; Model 6:** Adjusted for intrauterine growth restriction.

## Discussion

There was a reduction of PMR, NMR and IMR in all three cities and a trend to a decelerated reduction of the mortality coefficients during the most recent period in Ribeirão Preto, and even stagnation of the indicators in Pelotas (1993–2004) and of stillbirths in São Luís. The sociodemographic variables were those that most explained the reduction of the NMR and IMR in Ribeirão Preto (1978/79–1994) and São Luís, considering the increase of OR when these variables were included in the model. Unfavorable changes in gestational age were observed in all cities and periods, contributing to a lower reduction of NMR and IMR (hence the reduction of OR following the inclusion of this variable).

From the second to the third period, there was a deceleration of the reduction in IMR in Ribeirão Preto, and stagnation in Pelotas. Barros *et al*.^25^, using Brazilian data, observed that the annual rate of IMR reduction was lower after 2000 compared to previous decades. It is possible that the interventions implemented for the reduction of IMR during the 1980 and 1990 decades exerted most of their effect up to 2000.

In Pelotas, the SBR showed the reduction of lowest magnitude from the first to the second period and stagnation from the second to the third period, continuing to have the highest rate among the three cities. The increased preterm birth rate, reported by Barros *et al*.^17^, may probably help explain part of this stagnation, with a negative effect against the improvement of the sociodemographic indicators and of the access to and coverage by the health services.

The PMR were reduced in all cities and periods studied, except in Pelotas from 1993 to 2004. However, in the latest periods, all cities still had a high PMR when compared to developed countries^26^. These data point to the importance of investments that would reduce maternal complications, as well as improve maternal and NB care from gestational to neonatal period^27^.

The difference in the reduction of the mortality indicators between the three cities underscore the effect of socioeconomic differences and of differences in access to health services and to medical technology^28^. Ribeirão Preto, the city with the best socioeconomic situation, with greater access to health services and medical technology^29^, was the city that succeeded in achieving the lowest mortality rates at the end of the study period.

The stagnation of the mortality rates in Pelotas from 1993 to 2004 is an alert to the need for greater monitoring, improved socioeconomic indicators and childbirth care. This city has gone through an economic decline caused by the reduction of industrial activity and has become increasingly poorer, probably contributing to the stagnation of the indicators^30^. The gross domestic product per capita in Pelotas was 9% higher than the national mean in 1982, with a fall to a 36% lower value than the mean in 2004^31^.

The improvements in sociodemographic characteristics exceeded the impact of negative changes in the distribution of gestational age, leading to a reduction of NMR and IMR in Ribeirão Preto (1978/79–1994) and São Luís. Increased maternal schooling, reduced poverty, increased urbanization and a fall in the fecundity rate^13,25^ are advances that contributed to the reduction of socioeconomic inequalities and, combined with public policies that expanded the access to health care, are pointed out as major factors responsible for the decline of infant mortality in Brazil^13,32,33^.

The reduction of IUGR in Ribeirão Preto (1994–2010) and in Pelotas (1982–1993) contributed to increase the OR when this variable was included in the adjusted model. This result indicates that IUGR helped explain the reduction of NMR and IMR in these periods.

Gestational age was the variable that most contributed to slowing or even stopping the decline of the NMR and IMR. In Ribeirão Preto from 1994 to 2010, the reduction of IUGR explained the reduction of NMR and IMR, even though the reduction of gestational age and unfavourable changes in sociodemographic variables prevented an even greater decline in NMR and IMR. In the US, an increase in preterm and low birth weight rate was responsible for the stagnation of NMR and IMR from 2000 to 2005^34^ similar to that observed in the present study.

Improved prenatal care and advances in health technologies have increased the survival of extremely preterm and very low birth weight babies. However, the use of medical interventions such as elective caesarean deliveries has had a negative impact on NB health, generating an increase in the preterm birth rate^19,35–37^. In addition, several studies have reported a higher NMR and IMR among late preterm babies^37–39^, with a higher mortality risk among babies of the same gestational age born by elective caesarean delivery^40^. On this basis, we emphasize the importance of implementing health policies that discourage unnecessary caesarean deliveries, especially elective ones and those performed before 37 weeks of gestation.

It is probable that postnatal variables described in the literature may have been also responsible for the observed declines because of their influence on the fall of NMR and IMR. Particularly outstanding among these factors are the almost universal coverage of vaccination, the increase in the average duration of breastfeeding^41^, the expressive reduction of infant mortality due to diarrhoea^42^, a greater access to neonatal intensive care units^43^, the impact of the Family Health Strategy^44^ and of programs of nutrient supplementation and fortification^45^, among others. However, it was not possible to observe their effect in the present study, because not all cohorts collected these data.

The main strong points of the present study are the combination of the data on eight birth cohorts located in regions with contrasting socioeconomic characteristics and the presentation of changes in different indicators. The comparisons were made between pairs of years since the evolution of the indicators was not linear. In addition, all cohorts are population-based, with a reduced percentage of losses and used similar methods.

Some limitations of this study should be pointed out, such as the absence of variables for the postnatal period and data about changes in postnatal health services, as well as the differences in the periods at which each cohort was started. It is important to note that the last Pelotas cohort is six years older than the last ones of the other two cities. In addition, the definition of stillbirth considered in Pelotas differed from that considered in São Luís and Ribeirão Preto. This difference in criterion, in addition to compromising the comparability of rates between the cities, possibly underestimated the PMR and SBR in Pelotas compared to the other cities.

## Conclusion

Most of the reduction in NMR and IMR was explained by the improvement of sociodemographic variables and by the reduction of IUGR rates. Nevertheless, NMR and IMR are still high compared to high-income countries and the decreasing rates have shown deceleration. NMR and IMR might have shown an even greater decrease were it not for the decline observed in gestational age.

Our study presents the effect of important risk factors on infant and neonatal mortality rates, allowing for better targeting of public health policies aimed at reducing infant mortality indicators. Factors such as the increase of family income and maternal schooling and the decreased IUGR contributed to the reduction of IMR and NMR. On the other hand, the reduction in GA prevented IMR and NMR from reaching even lower rates. Therefore, public policies focused on increasing family income and maternal schooling are necessary, as well as actions aimed at reducing the prevalence of IURG and preventing the reduction of gestational age.

Future studies could be of interest to find out other risk factors, especially postnatal, that determine the indicators of child mortality in middle-income countries.

### Ethical approval

“All procedures performed in studies involving human participants were in accordance with the ethical standards of the institutional and/or national research committee”. For the older cohorts we obtained the approval of an Internal Committee of the respective Schools of Medicine (Ethics Committee of the University Hospital of the Federal University of Maranhão, Ethics Committee of the Hospital of the Clinics of the Federal University of São Paulo, Ethics Committee of the Federal University of Pelotas) and verbal informed maternal consent. All other cohorts were approved by the Research Ethics Committees of the teaching institutions to which they were linked and the mothers gave written informed consent before interview.

## Supplementary information

Supplementary table 1. Comparison of the socioeconomic, demographic, life style and health service characteristics in Ribeirão Preto (1978/79, 1994 and 2010).

## Data Availability

The datasets generated during and/or analysed during the current study are not public but are available from the corresponding author on reasonable request.
