# Author Correction: Changes in Infant and Neonatal Mortality and Associated Factors in Eight Cohorts from Three Brazilian Cities

**DOI:** 10.1038/s41598-020-66188-2

**Published:** 2020-06-08

**Authors:** Carolina A. Carvalho, Antônio A. M. da Silva, César Victora, Marcelo Goldani, Heloísa Bettiol, Erika Barbara Abreu Fonseca Thomaz, Fernando Barros, Bernardo L. Horta, Ana Menezes, Viviane Cardoso, Ricardo Carvalho Cavalli, Iná Santos, Rosângela F. L. Batista, Vanda Maria Simões, Marco Barbieri, Aluisio Barros

**Affiliations:** 1 Federal Institute of Maranhão; Federal University of Maranhão, Post Graduate Program in Collective Health, Rua Barão de Itapary, nº 155, Centro, Zipcode: 65.020-070, São Luís, MA Brazil; 2 Federal University of Maranhão, Post Graduate Program in Collective Health, Rua Barão de Itapary, nº 155, Centro, Zipcode: 65.020-070, São Luís, MA Brazil; 3 Federal University of Pelotas, Post Graduate Program in Epidemiology, Pelotas, Brazil; 4 Federal University of Rio Grande do Sul, Department of Pediatrics, Porto Alegre, Brazil; 5 University of São Paulo, Department of Puericulture and Pediatrics, Ribeirão Preto, Brazil; 6 Catholic University of Pelotas, Post-Graduate Program in Health and Behavior and Federal University of Pelotas, Post Graduate Program in Epidemiology, Pelotas, Brazil; 7 University of São Paulo, Department of Gynecology and Obstetrics, Ribeirão Preto, Brazil

Correction to: *Scientific Reports* 10.1038/s41598-020-59910-7, published online 24 Fabruary 2020

In the original version of this Article the author Erika Barbara Abreu Fonseca Thomaz, was incorrectly given as Erika Barbara Thomaz. This has now been corrected in the PDF and HTML versions of the Article, and in the accompanying Supplementary Information file.

As a result, in the Author Contributions statement

“All authors (Carolina Abreu de Carvalho, Antônio AM da Silva, César Victora, Marcelo Goldani, Heloísa Bettiol, Erika Barbara Thomaz, Fernando Barros, Bernardo L. Horta, Ana Menezes, Viviane Cardoso, Ricardo Carvalho Cavalli, Iná Santos, Rosângela FL Batista, Vanda Maria Simões, Marco Barbieri, Aluisio Barros) have made substantial contributions to all of the following: (1) the conception and design of the study, or acquisition of data, or analysis and interpretation of data, (2) drafting the article or revising it critically for important intellectual content, (3) final approval of the version to be submitted.”

now reads:

“All authors (Carolina Abreu de Carvalho, Antônio AM da Silva, César Victora, Marcelo Goldani, Heloísa Bettiol, Erika Barbara Abreu Fonseca Thomaz, Fernando Barros, Bernardo L. Horta, Ana Menezes, Viviane Cardoso, Ricardo Carvalho Cavalli, Iná Santos, Rosângela FL Batista, Vanda Maria Simões, Marco Barbieri, Aluisio Barros) have made substantial contributions to all of the following: (1) the conception and design of the study, or acquisition of data, or analysis and interpretation of data, (2) drafting the article or revising it critically for important intellectual content, (3) final approval of the version to be submitted.”

