# Supplementary material for: Changes in Infant and Neonatal Mortality and Associated Factors in Eight Cohorts from Three Brazilian Cities
Source: Sci Rep. 2020 Feb 24;10:3249. doi: 10.1038/s41598-020-59910-7 (PMC7039903; doi:10.1038/s41598-020-59910-7)
Supplement: Supplementary file 1 — Supplementary table 1. Comparison of the socioeconomic, demographic, life style and health service characteristics in Ribeirão Preto (1978/79, 1994 and 2010). [file 41598_2020_59910_MOESM1_ESM.pdf]

CHANGES IN INFANT AND NEONATAL MORTALITY AND ASSOCIATED FACTORS IN EIGHT COHORTS FROM THREE BRAZILIAN CITIES

Carolina Carvalho, Antônio AM da Silva, César Victora, Marcelo Goldani, Heloísa Bettiol, Erika Barbara Thomaz, Fernando Barros, Bernardo L. Horta, Ana Menezes, Viviane Cardoso, Ricardo Carvalho Cavalli, Iná Santos, Rosângela FL Batista, Vanda Maria Simões, Marco Barbieri, Aluisio Barros

Supplementary table 1. Comparison of the socioeconomic, demographic, life style and health service characteristics in Ribeirão Preto (1978/79, 1994 and 2010).

	RIBEIRÃO PRETO					RIBEIRÃO PRETO				
Variables	1978/79		1994		p-value	1994		2010		p-value
**Maternal schooling (years)**	%	n	%	n	<0.001	%	n	%	n	<0.001
≥12	10.1	685	13.6	371		13.6	371	23.5	1795	
9 to 11	13.5	911	23.2	632		23.2	632	50.5	3854	
5 to 8	25.1	1694	39.1	1067		39.1	1067	22.0	1682	
0 to 4	51.3	3468	24.2	659		24.2	659	3.9	301	
**Marital status**					<0.001					0.119
With a partner	93.1	6416	87.2	2446		87.2	2446	86.0	6665	
Without a partner	6.9	476	12.8	359		12.8	359	14.0	1083	
**Newborn Sex**					0.644					0.228
Female	48.6	3387	49.1	1435		49.1	1435	50.4	3928	
Male	51.4	3582	50.9	1487		50.9	1487	49.6	3863	
**Maternal age (years)**					<0.001					<0.001
20-34	77.5	5351	73.1	2130		73.1	2130	74.4	5803	
≥35	8.7	597	9.4	274		9.4	274	12.8	998	
<20	13.9	957	17.6	512		17.6	512	12.8	996	
**Maternal smoking**					<0.001					<0.001
No	71.1	4766	78.3	2208		78.3	2208	88.2	6840	
Yes	28.9	1933	21.7	613		21.7	613	11.8	915	
**Parity**					<0.001					<0.001
1	36.2	2445	41.0	1187		41.0	1187	50.0	3875	
2 to 4	52.7	3561	53.2	1540		53.2	1540	46.8	3626	
≥5	11.1	751	5.8	168		5.8	168	3.2	244	
**Type of pregnancy**					0.555					0.596
Single	97.96	6827	97.8	2858		97.8	2858	97.6	7611	
Multiple	2.04	142	2.2	65		2.2	65	2.4	187	
**Prenatal care**					<0.001					<0.001
Yes	91.5	5555	97.1	2587		97.1	2587	98.6	7022	
No	8.5	516	2.9	77		2.9	77	1.4	102	
**Type of hospital**					<0.001					<0.001
Public	92.8	6219	63.2	1757		63.2	1757	55.6	4312	
Private	7.2	482	36.8	1022		36.8	1022	44.4	3449	
**Type of delivery**					<0.001					<0.001
Vaginal	69.3	4825	48.6	1419		48.6	1419	41.1	3206	
Cesarean	30.7	2140	51.5	1504		51.5	1504	58.9	4587	
**Gestational age**					<0.001					0.639
Mean	38.9	6969	38.3	2923		38.3	2923	38.2	7798	
**IUGR***					0.005					<0.001
No	85.1	5914	82.5	2407		82.5	2407	85.4	6663	
Mild or moderate	10.0	691	11.4	333		11.4		10.1	784	
Severe	4.9	342	6.0	176		6.0		4.5	351	

*Intrauterine growth restriction

Supplementary table 2. Comparison of the socioeconomic, demographic, life style and health service characteristics in Pelotas (1982, 1993 and 2004) and São Luís (1997/98 and 2010).

	PELOTAS					PELOTAS					SÃO LUÍS				
**Variables**	**1982**		**1993**		**p-value**	**1993**		**2004**		**p-value**	**1997/98**		**2010**		**p-value**
**Maternal schooling (years)**	%	n	%	n	<0.001	%	n	%	n	<0.001	%	n	%	n	<0.001
≥12	14.1	849	8.2	427		8.2	427	9.9	422		4.8	122	15.3	785	
9 to 11	10.9	657	17.6	923		17.6	923	32.9	1395		35.4	896	57.7	2955	
5 to 8	41.6	2495	46.2	2424		46.2	2424	41.5	1761		42.6	1079	22.8	1168	
0 to 4	33.4	2003	28.0	1468		28.0	1468	15.7	666		17.3	437	4.2	213	
**Marital status**					<0.001					<0.001					<0.001
With a partner	91.8	5511	87.6	4600		87.6	4600	83.4	3577		75.1	1908	80.4	4192	
Without a partner	8.2	495	12.4	649		12.4	649	16.6	710		24.9	632	19.6	1020	
**Newborn Sex**					0.063					0.027					0.001
Female	48.6	2923	50.4	2645		50.4	2645	48.1	2061		45.1	1147	49.0	2551	
Male	51.4	3086	49.6	2603		49.6	2603	51.9	2222		54.9	1394	51.0	2657	
**Maternal age (years)**					0.001					<0.001					<0.001
20 to 34	74.6	4485	71.6	3756		71.6	3756	67.6	2897		66.5	1688	73.6	3838	
≥35	10.1	604	11.0	577		11.0	577	13.5	577		4.2	107	7.9	413	
<20	15.3	921	17.4	915		17.4	915	18.9	811		29.3	744	18.4	961	
**Maternal smoking**					0.011					<0.001					<0.001
No	64.4	3868	66.6	3497		66.6	3497	72.4	3104		94.0	2388	95.9	4999	
Yes	35.7	2143	33.4	1752		33.4	1752	27.6	1183		6.0	153	4.1	213	
**Parity**					0.107					<0.001					0.070
1	34.8	2092	35.1	1843		35.1	1843	39.3	1684		48.6	1236	47.2	2460	
2 to 4	52.6	3158	53.5	2810		53.5	2810	50.7	2171		47.0	1193	49.2	2563	
≥5	12.7	760	11.4	596		11.4	596	10.1	431		4.4	112	3.6	188	
**Type of pregnancy**					0.520					0.086					0.975
Single	98.3	5909	98.5	5168		98.5	5168	98.0	4201		98.0	2491	98.0	5110	
Multiple	1.7	102	1.5	81		1.5	81	2.0	86		2.0	50	2.0	102	
**Prenatal care**					0.433					<0.001					<0.001
Yes	94.9	5677	95.2	4987		95.2	4987	98.0	4025		91.5	2298	97.8	4843	
No	5.1	306	4.8	251		4.8	251	2.0	81		8.5	213	2.2	107	
**Type of hospital**					0.572					<0.001					<0.001
Public	90.9	5465	90.6	4756		90.6	4756	81.2	3475		89.2	2266	84.2	4388	
Private	9.1	546	9.4	493		9.4	493	18.8	806		10.8	275	15.8	823	
**Type of delivery**					0.001					<0.001					<0.001
Vaginal	72.4	4352	69.5	3647		69.5	3647	54.8	2350		66.2	1683	52.6	2744	
Cesarean	27.6	1659	30.5	1602		30.5	1602	45.2	1937		33.8	858	47.4	2468	
**Gestational age**					<0.001					<0.001					<0.001
Mean	39.2	4747	38.0	5171		38.0	5171	38.4	4262		38.5	2541	38.3	5212	
**IUGR***					<0.001					<0.001					0.027
No	76.9	4617	84.5	4420		84.5	4420	81.7	3493		84.8	2145	86.6	4485	

Mild or moderate	14.2	849	11.2	588	11.2	588	12.6	540	10.1	255	9.5	494
Severe	8.9	536	4.3	224	4.3	224	5.7	244	5.1	129	3.9	200

*Intrauterine growth restriction
